# Validation of key components in designing a social skills training content using virtual reality for high functioning autism youth—A Fuzzy Delphi method

**DOI:** 10.1371/journal.pone.0301517

**Published:** 2024-04-04

**Authors:** Mohd Akif Farhan Ahmad Basri, Wan Salwina Wan Ismail, Norazlin Kamal Nor, Noorlaili Mohd Tohit, Mohammad Nazir Ahmad, Nur Saadah Mohamad Aun, Tuti Iryani Mohd Daud

**Affiliations:** 1 Department of Psychiatry, Faculty of Medicine, Hospital Canselor Tuanku Muhriz, Universiti Kebangsaan Malaysia, Wilayah Persekutuan Kuala Lumpur, Malaysia; 2 Department of Paediatrics, Faculty of Medicine, Hospital Canselor Tuanku Muhriz, Universiti Kebangsaan Malaysia, Wilayah Persekutuan Kuala Lumpur, Malaysia; 3 Department of Family Medicine, Faculty of Medicine, Hospital Canselor Tuanku Muhriz, Universiti Kebangsaan Malaysia, Wilayah Persekutuan Kuala Lumpur, Malaysia; 4 Institute of IR4.0, Akademia Siber Teknopolis, Universiti Kebangsaan Malaysia, Selangor, Malaysia; 5 Centre for Research in Psychology and Human Well-Being, Faculty of Social Sciences and Humanities, Universiti Kebangsaan Malaysia, Selangor, Malaysia; The University of Sydney, AUSTRALIA

## Abstract

The use of virtual reality in social skills training for high functioning autism spectrum disorder (HFASD) youth has been found to be engaging and enjoyable. Despite the promising results, previous literature indicates that there has been no consensus on the social skills target in the training content. There is also limited research on how evidence-based strategies like cognitive and behaviour techniques are instantiated into the VR environment to teach social skills. The aim of this study is to determine the key components to design a social skills training content using virtual reality for youths with HFASD. The Fuzzy Delphi method (FDM) was used to obtain expert consensus on social skills difficulties and cognitive behavioral techniques included in the content in three phases. In phase 1, a questionnaire was developed from in-depth interviews and scientific literature review. The in-depth interviews were conducted with 13 HFASD youth, 7 parents and 6 experts. In phase 2, 3 experts rated the relevance of the items in the questionnaire using an item-level content validity index (I-CVI) assessment. In phase 3, the questionnaire was distributed to 10 experts to rate their level of agreement on each component using a 7-point Likert scale. Components that received a value above 75%, threshold value (d) ≤ 0.2, fuzzy score (A) ≥ α - cut value = 0.5 and higher rank based on defuzzification score were prioritized to be included in the content. Items that received higher expert consensus on social skills difficulties included assessing non-verbal responses, initiating, maintaining, and leaving conversations, emotional difficulties and difficulties in perspective taking. Cognitive and behavioral techniques that received higher expert consensus were psychoeducation, modelling, relaxation techniques, reinforcements, and perspective-taking questions. These key components can be used as a framework for the development of virtual learning content for social skills training in future studies.

## Introduction

Autism spectrum disorder (ASD) ranges in a spectrum from low functioning (LFASD) to high functioning (HFASD) individuals. Even though the terms “low” and “high” are not officially used in the DSM-5, published past literature and clinicians in the field usually refer to these terms to indicate differences in behaviors, cognition, language, and social communication [1]. It has been suggested that around 44% of ASD individuals have IQ above 85, which is used as a parameter indicative of HFASD [2]. Even though those with HFASD have normal cognitive ability, social skills difficulties remain a central deficit [3].

Virtual reality (VR) has been recognized as a promising new intervention application that can improve social skills in ASD individuals, mainly due to the enjoyability and engaging aspects of VR that motivates ASD users to learn [4]. Previous studies using VR interventions found improvements in social communication [5], social attention [6], play skills [7], emotional regulation skills [8], and social cognition [9] among individuals with ASD in different age groups.

Despite the promising results in earlier studies, there has been a lack of consensus and inconsistency on the priority of which social skills difficulty to be targeted in the virtual learning content [4, 10–12]. Thai and Nathan-Roberts [11] concluded that “*there is a large variation in which specific social skills should be targeted*” (p. 1471) in their systematic review of 12 intervention studies on social skills using VR. Similarly, Mesa-Gresa et al. [10] found a large variety of social skills targets in their systematic review of 31 articles. The social skills targets across studies varied from improving emotion recognition [13–16], communication skills [17], social understanding [18], collaboration and reciprocity in behavior and communication [19], performance in facial affect and recognition [20], social responsiveness and executive functioning skills [21], as well as nonverbal and gesturing communication [22]. Hence, determining which social skills target to be prioritized in designing the learning content for social skills training using VR is important.

There is also a dearth of research on the “what” and “how” evidence-based techniques are used to teach social skills in VR form [4, 10–12, 23, 24]. Parsons and Mitchell [24] suggested that contents in VR can be designed to instantiate both behavioral and cognitive approaches to learning to bridge a user-centered design with evidence-based strategies. Even though it is widely known that cognitive and behavioral techniques are effective in physical social skills training [23], there has been a lack of studies exploring how these techniques are instantiated in the form of VR [4, 10–12, 24]. Hence, determining which cognitive and behavioral techniques can be utilized in training social skills using VR is important in designing the learning content.

This study was conducted to determine expert consensus on the selection of social skills difficulties and cognitive and behavioral techniques to be included in the social skills training content using VR for HFASD youths.

## Methodology

The Fuzzy Delphi method (FDM) was used to obtain expert consensus on the key components to be included in the content. The FDM is an adaptation from the classic Delphi method, in which it adds a set of fuzzy numbering sets while maintaining the Delphi method itself [25]. The FDM reduces the cycle process to avoid loss of data, leading to a higher economic efficacy in time and cost [26]. The FDM has been widely used to validate the components for training contents due to its ability to obtain the value of fuzzy scores in the form of ranking that can be used as a determinant and priority of an element according to expert consensus [27–29]. This study consisted of three phases:

### Phase 1

The objective in Phase 1 was to explore the needs analysis of the social skills training content. This was conducted using in-depth interviews and scientific literature review.

The in-depth interviews were conducted with 13 HFASD youths, 7 parents and 6 experts, chosen through a *purposive maximal variation* sampling technique. We purposely and intentionally selected these participants with specific criteria to strengthen data triangulation from different perspectives. The HFASD youth shared their perspectives from lived experience, parents shared their experience raising and living with HFASD individuals, while the experts shared their experience from a professional perspective. These three groups were chosen as they were identified as key stakeholders in the process of designing a user-friendly learning content [30]. The objective of the in-depth interviews was to explore the social skills difficulties that are faced by HFASD and the cognitive behavioural techniques that can help them to learn social skills in everyday life.

The scientific literature review was explored using four primary databases, which are PubMed, APA PsycINFO, Scopus and Google Scholar using keywords such as (autism OR ASD OR Asperger) AND (social skills OR social communication skills) AND (virtual reality OR VR). Recent articles from January 2010 to December 2022 were exclusively reviewed in the analysis due to the fast-paced advancement in technology in this century, as technology plays a crucial role in VR-related studies. The objective of this is to explore the social skills difficulties and cognitive behavioral techniques that have been used in social skills training using virtual reality.

The inclusion criteria for the groups were:

### HFASD Youths

Diagnosed with HFASD by a mental health professional or developmental pediatrician, aged between 15 to 24 years, and able to converse in English or Malay.

### Parents

Mother, father, or caregiver of a diagnosed youth with HFASD, and able to converse in English or Malay.

### Experts

A clinical psychologist, psychiatrist, speech therapist, or occupational therapist with a minimum of five years of professional experience conducting social skills training with HFASD youth, familiar with cognitive and behavior techniques, and able to converse in English or Malay.

### Procedure

Ethics approval was granted from the National University of Malaysia Research Ethics Committee on 11^th^ March 2021 (Ref. No: UKM PPI/111/8/JEP-2021-092). Potential participants were contacted via telephone. They were informed about the study and invited for the interview once they fulfilled the inclusion criteria. Written informed consent for participants above 18 years was obtained via email. For participants under 18 years, written consent was obtained from the parents or guardians. Participants were recruited between April to August 2021. The time and date for the interview was arranged according to the participants’ convenience. All interviews were conducted online due to movement restriction orders during the peak of COVID-19 period in Malaysia. The main language of interview was English, Malay or combination of both, based on the preference of the participants. A semi-structured interview protocol was used. The flow of the questions was mainly influenced by the participants’ cues and context. The questions were mainly to explore the social skills difficulties faced by HFASD youths as well as the cognitive and behavioral techniques that helped in improving their social skills. All interviews were recorded and saved in Health Insurance Portability and Accountability Act 1996 (HIPAA) compliant applications. All authors had access to information that identified the participants during and after data collection. The privacy and confidentiality of the participants’ background information were protected as all participants were identified as initials in recordings, transcripts, analysis, and data reporting.

### Data analysis

The recorded interview videos were transcribed verbatim and then transferred to Nvivo 12. The qualitative analysis begins with open coding in which the transcripts are read line by line and participants’ exact phrases are used as initial codes. Each of the codes are compared with other data in the process of developing a theme. Codes that match each other were categorized under the same themes, while codes that did not match were categorized into a new theme. Similar themes were then analyzed whether it fits into a broader categorization. This approach follows an inductive method.

Then, we consistently reviewed the data to identify any quotes that may have been overlooked and should have been incorporated into the established themes and sub-themes. This approach follows a deductive method. This process continues until all relevant quotes from the transcripts have been categorized under themes or subthemes and there are no longer new themes that emerge from the transcripts.

The qualitative data was triangulated between each of the groups (HFASD, parents and experts). The themes and sub-themes were then verified with the scientific literature review findings. The themes and sub-themes that had support from literature review were converted into items in a questionnaire, which was then reviewed in Phase 2.

### Phase 2

The objective in Phase 2 was to review the relevance of items in the questionnaire developed in Phase 1 using an item-level content validity index (I-CVI) assessment. The I-CVI assessment is a critical step in enhancing the validity of the questionnaire [31]. Three experts were chosen by purposive sampling technique, in accordance with Lynn [32] who suggested 3 experts as the minimum requirement. These experts were selected based on the following criteria:

- A clinical psychologist, psychiatrist, speech therapist, or occupational therapist with a minimum of five years of professional experience conducting social skills training with HFASD youths.
- Familiar with cognitive and behavior techniques
- Able to converse in English or Malay.’
- Did not participate in the previous phase of this study.

### Procedure

Potential participants were contacted via telephone. They were informed about the study and invited to become an expert assessor once they fulfilled the inclusion criteria. Written informed consent was obtained via email. Participants were recruited between January to March 2022.The questionnaire developed in Phase 1 was distributed to the three experts. They rated the relevance of each item according to a 4-point ordinal scale: 1 = not relevant, 2 = somewhat relevant, 3 = quite relevant, 4 = highly relevant Items rated 3 or 4 are given a value of 1.00, while items rated as 1 or 2 are given a value of 0 [31].

### Data analysis

The value of I-CVI was calculated using the formula; I-CVI = n/N, in which ‘n’ is the number of experts agreed and ‘N’ is the sum of evaluator. Only items with the value of 1.00, which means that all three experts agree on the relevance of the item (rated 3 or 4), were accepted [32]. Items that received scores below 1.00 (rated 1 or 2 by at least 1 expert) were omitted. Past literature states that when the experts are fewer than 5 people, the I-CVI value must be 1.00 –that means, all experts must agree that the item is relevant [32]. The questionnaire containing the items that were accepted in this phase was used in the Fuzzy Delphi method in Phase 3.

### Phase 3

The objective in Phase 3 was to determine an expert consensus on the selection of social skills difficulties and cognitive behavioral techniques to be included in the social skills training content using VR. Ten experts chosen by purposive sampling were involved in the process of Fuzzy Delphi method, consistent with suggestions from previous literature [33]. The inclusion criteria of experts in this phase were:

- Clinical psychologist, psychiatrist, speech therapist or occupational therapist with a minimum of five years of professional experience conducting social skills training with HFASD youth.
- Familiar with cognitive behavior techniques.
- Able to converse in English or Malay.
- Did not participate in the previous phases of this study.

### Procedure

Potential participants were contacted via telephone. They were informed about the study and invited to become an expert assessor once they fulfilled the inclusion criteria. Written informed consent was obtained via email. Participants were recruited between June to July 2022. The questionnaire developed and reviewed in the previous phases were distributed to the experts via email, who rated their level of agreement on the items using a 7-point Likert scale, ranging from “Strongly Disagree = 1” to “Strongly Agree = 7” as shown in Table 1.

**Table 1:** Seven-point Likert scale and fuzzy scale.

7-point Likert Scale	Likert Scale	Fuzzy Scale		
**Strongly Agree**	7	0.9	1	1
**Agree**	6	0.7	0.9	1
**Somewhat Agree**	5	0.5	0.7	0.9
**Neutral**	4	0.3	0.5	0.7
**Somewhat Disagree**	3	0.1	0.3	0.5
**Disagree**	2	0	0.1	0.3
**Strongly Disagree**	1	0	0	0.1

### Data analysis

1. Step 1: Insert Likert scales into Microsoft Excel
  All the expert’s responses were inserted into a Microsoft Excel template [34–37] to analyze the fuzzy delphi scores.
2. Step 2: Convert the likert scales into triangular fuzzy numbers as suggested in Table 1. This conversion was conducted automatically by Microsoft Excel [34–37].
3. Step 3: Determine fuzzy average values from fuzzy scale.
  The fuzzy average values, m = (m1, m2, m3) was calculated automatically using the Microsoft Excel template [34–37].
4. Step 4: Specify the threshold value, d-construct.
  This was calculated automatically using the Microsoft Excel template [34–37]. The vertex method was used to calculate the distance between two fuzzy numbers $\tilde{m}=(m_{1},m_{2},m_{3})\;and\;\tilde{n}=(n_{1},n_{2},n_{3})$, as computed by Microsoft Excel using the following formula [34–37]:
$$d(\tilde{m},\tilde{n})=\sqrt{\frac{1}{3}[{\left(m_{1}-n_{1}\right)}^{2}+{\left(m_{2}-n_{2}\right)}^{2}+{\left(m_{3}-n_{3}\right)}^{2}]}$$
  An expert agreement is reached if the distance between the average value and the experts’ evaluation of data is smaller or equal to the threshold value (d) 0.2. If it was higher, the item was rejected [34–37].
5. Step 5: Gain a 75% consensus among the experts. This was calculated automatically using the Microsoft Excel template [34–37].
6. Step 6: Defuzzification process.

The defuzzification process, which is a process used to obtain the ranking of each item in the survey, was conducted. The formula for calculating the defuzzification value was A = (1/3) * (m1 + m2 + m3). The value that was used in defuzzification is the α-cut, which was the median for 0 and 1, α-cut = (0+1)/2 = 0.5. Items with a value of α-cut below 0.5 indicated a rejection, and items with a value above 0.5 indicated acceptance of expert consensus. The value of defuzzification also indicates the ranking for each item. A higher value provided a higher ranking, indicating a higher importance of the item. This was calculated automatically using the Microsoft Excel template [34–37].

Overall, Items that received a threshold value (d) ≤ 0.2, value above 75%, fuzzy score (A) ≥ α - cut value = 0.5 and higher rank based on defuzzification score were prioritized to be included in the content.

## Results

### Phase 1

#### Profile of participants

As shown in Table 2, 13 HFASD youths, 7 parents and 6 experts were involved in the in-depth interview. The gender proportion was equal. In terms of race, 65.4% (n = 17) were Malays, 24% (n = 6) Chinese and 12% (n = 3) Indians. The mean age of the HFASD youth was 19.77 years (SD = 2.74), ranging between 15 to 24 years old. In the parents’ group, the mean age was 48.29 (SD = 5.38), ranging between 42 to 56 years of age. The six experts included four clinical psychologists, one child and adolescent psychiatrist, and one speech language therapist.

**Table 2:** Profile of participants in Phase 1.

Participant	Group	Gender	Age	Race	Years of Working Experience
**1**	HFASD	Female	19	Malay	
**2**	HFASD	Male	20	Malay	
**3**	HFASD	Male	21	Malay	
**4**	HFASD	Male	22	Malay	
**5**	HFASD	Female	21	Chinese	
**6**	HFASD	Male	21	Chinese	
**7**	HFASD	Male	16	Malay	
**8**	HFASD	Male	24	Malay	
**9**	HFASD	Male	15	Malay	
**10**	HFASD	Male	23	Malay	
**11**	HFASD	Male	16	Indian	
**12**	HFASD	Male	20	Malay	
**13**	HFASD	Female	19	Malay	
**14**	Parent	Male	42	Indian	
**15**	Parent	Female	44	Malay	
**16**	Parent	Female	47	Malay	
**17**	Parent	Male	56	Malay	
**18**	Parent	Female	44	Malay	
**19**	Parent	Female	52	Chinese	
**20**	Parent	Female	53	Chinese	
**21**	Expert	Female	50	Malay	>10
**22**	Expert	Male	31	Chinese	>5
**23**	Expert	Female	42	Malay	>5
**24**	Expert	Female	40	Malay	>5
**25**	Expert	Female	38	Chinese	>10
**26**	Expert	Female	36	Indian	>5

### Results analysis

The extensive literature review and in-depth interviews results on social skills difficulties identified 5 major themes and 19 sub-themes as shown in Table 3. The major themes were social cognition, social communication skills, social emotions, social language, and stereotyped social behaviors. The results on cognitive and behaviour techniques helpful in training social skills in ASD identified 6 cognitive strategies and 9 behaviour strategies. The sub-themes were converted into items in a questionnaire and reworded in a phrase that would assist the experts to understand better, as shown in Table 5.

**Table 3:** Themes and sub-themes identified in Phase 1.

Domains	Themes	Sub-themes	Item No.
Social Skills Difficulties	Social Cognition	Focus	SCG1
		Insecurity	SCG2
		Motivation	SCG3
		Perspective taking	SCG4
		Self-Awareness	SCG5
	Social Communication	Acknowledge names & titles of respect	SCM6
		Saying appropriate words	SCM7
		Assessing non-verbal response	SCM8
		Initiating conversation	SCM9
		Maintaining & leaving conversation	SCM10
		Physical boundaries	SCM11
		Voice tone	SCM12
	Social Emotions	Social anxiety	SEM13
		Expressing emotions	SEM14
	Social Language	Language barrier	SLG15
		Understanding & expressing pragmatics	SLG16
	Stereotyped Social Behaviors	Eye contact	SSB17
		Rigidity	SSB18
		Self-facial expression	SSB19
Cognitive & Behavior Techniques	Cognitive	Perspective taking	CBT1
		Relaxation techniques	CBT2
		Self-reflect	CBT3
		Self-talk	CBT4
		Socratic questioning	CBT5
		Awareness of cognitive bias	CBT6
	Behavior	Modelling	CBT7
		Psychoeducation	CBT8
		Immediate feedback	CBT9
		Gradual exposure	CBT10
		Chaining	CBT11
		Shaping	CBT12
		Generalization	CBT13
		Skill Rehearsal	CBT14
		Reinforcements	CBT15

### Phase 2

As shown in Table 4, the three experts were a child and adolescent psychiatrist, a pediatrician, and a family medicine specialist. All of them had more than 10 years of working experience. Two of them were PhD holders while the other one had master’s degree as the highest level of education.

**Table 4:** Profile of participants in Phase 2.

Participant	Expertise	Gender	Level of Education	Years of Working Experience
**1**	Child & Adolescent Psychiatrist	Female	Masters	>10
**2**	Paediatrician	Female	PhD	>10
**3**	Family Medicine Specialist	Female	PhD	>10

### Results analysis

Sub-themes derived from Phase 1 of this study were converted into items in a questionnaire and reworded into phrases that would be easier to understand as shown in Table 5. Out of the 19 items in the social skills difficulties domain, 15 were accepted, and 4 were rejected. Out of 15 items in the cognitive and behavior techniques domain, 11 were accepted and 4 were rejected. In summary, items of 15 social skills difficulties and 11 cognitive behavior techniques that were rated as most relevant are retained in the questionnaire, which is used in the fuzzy delphi method in Phase 3.

**Table 5:** Analysis of I-CVI values.

Item No.	Item Label	X1	X2	X3	I-CVI Value	Item
						Status
SCG1	Difficulties focusing on the topic conversation	1	1	1	1.00	Accept
SCG2	Feels insecure to make friends	0	1	1	0.67	Reject
SCG3	Low levels of motivation	1	1	0	0.67	Reject
SCG4	Perceiving from another person’s perspective	1	1	1	1.00	Accept
SCG5	Self-awareness of social challenges	1	1	1	1.00	Accept
SCM6	Acknowledge names & titles of respect	1	1	1	1.00	Accept
SCM7	Knowing appropriate words to converse	1	1	1	1.00	Accept
SCM8	Assessing people’s non-verbal response	1	1	1	1.00	Accept
SCM9	Starting a conversation	1	1	1	1.00	Accept
SCM10	Maintaining & leaving a conversation	1	1	1	1.00	Accept
SCM11	Physical boundaries between people	1	1	1	1.00	Accept
SCM12	Inappropriate use of voice tone	1	1	1	1.00	Accept
SEM13	Social anxiety	1	1	1	1.00	Accept
SEM14	Expressing emotions	1	1	0	0.67	Reject
SLG15	Language barrier	0	0	1	0.33	Reject
SLG16	Understanding & expressing pragmatics	1	1	1	1.00	Accept
SSB17	Difficulties maintaining eye contact	1	1	1	1.00	Accept
SSB18	Rigidity in interest & topics to talk about	1	1	1	1.00	Accept
SSB19	Lack of facial expression	1	1	1	1.00	Accept
CBT1	Perspective taking questions	1	1	1	1.00	Accept
CBT2	Relaxation techniques	1	1	1	1.00	Accept
CBT3	Self-reflections	1	0	1	0.67	Reject
CBT4	Self-talk	1	1	1	0.67	Reject
CBT5	Socratic questioning	1	1	1	1.00	Accept
CBT6	Awareness of cognitive bias	1	1	1	1.00	Accept
CBT7	Modelling	1	1	1	1.00	Accept
CBT8	Psychoeducation	1	1	1	1.00	Accept
CBT9	Immediate feedback	1	1	1	1.00	Accept
CBT10	Gradual exposure	1	1	1	1.00	Accept
CBT11	Chaining	0	0	1	0.33	Reject
CBT12	Shaping	0	0	1	0.33	Reject
CBT13	Generalization	1	1	1	1.00	Accept
CBT14	Skill Rehearsal	1	1	1	1.00	Accept
CBT15	Reinforcements	1	1	1	1.00	Accept

*X = experts

### Phase 3

As shown in Table 6, the 10 experts in this phase included 5 clinical psychologists, 1 psychiatrist, 2 developmental psychologists, and 2 psychologists with expertise in digital content development. Most of them (70%, n = 7) had more than 10 years of working experience, while the others (30%, n = 3) had experience between 5 to 10 years. Half of the experts (50%, n = 5) had master’s degree as the highest level of education while the other half (50%, n = 5) were PhD holders.

**Table 6:** Profile of participants in Phase 3.

Participant	Expertise	Gender	Level of Education	Years of Working Experience
**1**	Psychiatrist	Female	Masters	>10
**2**	Clinical Psychologist	Female	Masters	>10
**3**	Clinical Psychologist	Female	PhD	5–10
**4**	Developmental Psychologist	Male	PhD	>10
**5**	Clinical Psychologist	Female	PhD	>10
**6**	Clinical Psychologist	Male	Masters	5–10
**7**	Psychology & Digital Content Development	Female	PhD	>10
**8**	Developmental Psychologist	Female	Masters	5–10
**9**	Clinical Psychologist	Female	Masters	>10
**10**	Psychology & Digital Content Development	Female	PhD	>10

### Results analysis

All 15 items on social skills difficulties met the criteria for item acceptance. As shown in Table 7, the threshold value was ≤ 0.2, expert consensus exceeded 75% and fuzzy score (A) was more than 0.5. These items were then sorted by ranking according to the value of fuzzy score.

**Table 7:** Fuzzy Delphi scores on social skills difficulties items.

	Social Skills Difficulties	Triangular Fuzzy Numbers		Defuzzification		
Item	Item	Threshold value, d	Expert Consensus, %	Fuzzy Score (A)	Position	Expert Consensus
No.						
SG1	Difficulties focusing on the topic conversation	0.111	96.12%	0.886	14	Accepted
SG4	Perceiving from another person’s perspective	0.089	100.00%	0.922	5	Accepted
SG5	Lack of self-awareness of social challenges	0.102	94.82%	0.911	9	Accepted
SCM6	Acknowledge names & titles of respect	0.107	96.12%	0.898	12	Accepted
SCM7	Knowing appropriate words to converse	0.109	96.12%	0.887	13	Accepted
SCM8	Assessing people’s non-verbal response	0.063	96.12%	0.937	1	Accepted
SCM9	Starting a conversation	0.076	100.00%	0.933	2	Accepted
SCM10	Maintaining and leaving conversation	0.076	100.00%	0.933	2	Accepted
SCM11	Being aware of physical boundaries between people	0.099	88.78%	0.916	8	Accepted
SCM12	Inappropriate voice tone	0.097	92.67%	0.918	7	Accepted
SEM13	Social anxiety	0.083	96.12%	0.927	4	Accepted
SLG16	Understanding & expressing pragmatics	0.111	96.12%	0.886	14	Accepted
SSB17	Difficulties maintaining appropriate eye contact	0.093	94.82%	0.920	6	Accepted
SSB18	Rigidity in the topics to talk about	0.102	94.82%	0.911	9	Accepted
SSB19	Lack of self-facial expression	0.102	94.82%	0.911	9	Accepted

As shown in Table 8, items that were rated as high-ranking were difficulties assessing non-verbal response of other people, starting, maintaining, and leaving conversation, social anxiety, understanding another person’s perspective, maintaining appropriate eye contact, inappropriate voice tone and being aware of physical distance between people when talking.

**Table 8:** Social skills difficulties items by ranking of fuzzy score.

Sort by Priority	Items–Social Skills Difficulties	Item No.
**1**	Assessing people’s non-verbal response	SCM8
**2**	Starting a conversation	SCM9
**2**	Maintaining and leaving conversation	SCM10
**4**	Social anxiety	SEM13
**5**	Perceiving from another person’s perspective	SG4
**6**	Difficulties maintaining appropriate eye contact	SSB17
**7**	Inappropriate voice tone	SCM12
**8**	Being aware of physical boundaries between people	SCM11
**9**	Lack of self-awareness of social challenges	SG5
**9**	Rigidity in the topics to talk about	SSB18
**9**	Lack of self-facial expression	SSB19
**12**	Acknowledge names & titles of respect	SCM6
**13**	Knowing appropriate words to converse	SCM7
**14**	Difficulties focusing on the topic of conversation	SG1
**14**	Understanding & expressing pragmatics	SLG16

The results of the Fuzzy Delphi analysis in cognitive behavior techniques are shown in Table 9. In general, all 11 items met all three criteria for item acceptance. The threshold value was ≤ 0.2, expert consensus exceeded 75% and fuzzy score (A) was more than 0.5.

**Table 9:** Fuzzy Delphi scores on cognitive behaviour items.

	Cognitive Behaviour Techniques	Triangular Fuzzy Numbers		Defuzzification		
Item	Item	Threshold value, d	Expert Consensus, %	Fuzzy Score (A)	Position	Expert Consensus
No.						
CBT1	Perspective taking questions	0.083	100%	0.927	7	Accepted
CBT2	Relaxation techniques	0.063	100%	0.937	1	Accepted
CBT5	Socratic Questioning	0.083	100%	0.927	7	Accepted
CBT6	Awareness of cognitive bias	0.083	98.78%	0.927	7	Accepted
CBT7	Modelling	0.063	100%	0.937	1	Accepted
CBT8	Psychoeducation	0.063	100%	0.937	1	Accepted
CBT9	Immediate feedback	0.076	98.78%	0.933	5	Accepted
CBT10	Gradual exposure	0.102	98.78%	0.911	11	Accepted
CBT13	Generalization of skills	0.089	96.88%	0.922	10	Accepted
CBT4	Skill rehearsal	0.076	100%	0.933	5	Accepted
CBT15	Reinforcements	0.063	100%	0.937	1	Accepted

These items were then sorted by ranking according to the value of fuzzy score, as shown in Table 10. Items that were rated at high rankings were psychoeducation, modelling, relaxation techniques, reinforcements, role plays, immediate feedback, skill rehearsal, perspective taking questions, Socratic questioning, as well as awareness on cognitive bias.

**Table 10:** Cognitive behavioural techniques by ranking of fuzzy score.

Sort by Priority	Items–Cognitive & Behavioural Techniques	Item No.
1	Psychoeducation	CBT8
1	Modelling	CBT7
1	Relaxation techniques	CBT2
1	Reinforcements	CBT15
5	Immediate feedback	CBT9
5	Skill rehearsal	CBT4
7	Perspective taking questions	CBT1
7	Socratic Questioning	CBT5
7	Awareness of cognitive bias	CBT6
11	Generalization of skill	CBT13
11	Gradual exposure	CBT10

## Discussion

The result of this study provides a clear framework and guideline for researchers, clinicians, and trainers to develop and implement social skills training content using VR which has been validated by expert consensus. The components in this framework could be used as a standardized design for future research on the development of social skills training contents using VR for HFASD youths. This design could ensure that the content meets the needs of the HFASD youths and improve levels of efficacy in training.

### Social skills difficulties

One interesting point from this finding was the importance of considering the management of difficult emotions in social skills training. The experts rated ‘social anxiety’ highly as a social skills difficulty and ‘relaxation techniques’ as a strategy to help manage emotions and enhance social skills practice. This is highly important to acknowledge as HFASD individuals commonly report high levels of anxiety and fear of rejection whenever they try to practice their social skills in the real-world environment [38]. Furthermore, the benefit of VR itself, which can simulate real-world social communication scenarios, can provide a safe space for ASD individuals to train themselves without fear of failure or rejection [38].

The experts also rated highly on assessing people’s non-verbal response, as well as difficulties in starting, maintaining, and leaving a conversation—all of which are fundamental challenges in individuals with ASD due to mind-blindness, which means they struggle to attribute mental states to others [39]. This was consistent with the focus of Program for the Education and Enrichment of Relational Skills (PEERS®), developed by Laugeson [40], a face-to-face manualized social skills program with the highest number of efficacy studies [41]. The reason this is important is that high-functioning autism youth often lack the knowledge of the typical steps followed by their neurotypical peers [26]. They are hesitant to engage in conversations because, when they do, they frequently face rejection, leading to emotional trauma and social isolation [42]. Furthermore, these social steps of assessing people’s non-verbal response, starting, maintaining, and leaving conversations are relevant to teach because they are versatile and can be applied in various contexts, such as school, college, the workplace, and family gatherings [40].

### Cognitive behavior techniques

Results are consistent with systematic review findings that one of the most used techniques of behavior skills is modelling, including video modelling [41]. Recent studies also show promising results on how video modelling can be instantiated in spherical video-based virtual reality (SVVR) for autistic users [43]. SVVR, also known as 360-degree videos or immersive videos, involves positioning users at the center of a spherical environment [44]. In this setup, users engage with the content and context by moving their head, as detected by a connected Head-Mounted Display (HMD). Recent studies indicate that there is some supporting evidence indicating that skills learned in SVVR through video modelling can be generalized to real-world situations [44–47].

Research has also shown preliminary data on how relaxation techniques can be incorporated into VR and the results are promising. For example, in a study by Meindl et al. [46], they explored the use of relaxation techniques within exposure therapy in SVVR to an adult with autism gradually confront his needle phobia. They developed a special SVVR program where the participant watched a 360-degree video in a VR headset, making it feel like he was in a doctor’s office. The researchers found that incorporating relaxation techniques in this SVVR method was better than regular exposure therapy because it could replicate real-life situations like doctors’ offices using affordable VR technology. Results indicated that the participant became more comfortable with medical procedures and different healthcare providers. Similarly, Maskey et al. [48] worked with eight autistic adults to assess a VR-based intervention for anxiety management. The participants underwent a session with a therapist to learn anxiety management techniques, followed by four 20-minute sessions in a virtual reality (VR) environment called the Blue Room, tailored to their specific anxiety triggers. The study found five out of eight participants showed lasting improvements in their real-life anxiety-related situations six months after the VR sessions. Both studies demonstrate the potential effectiveness of incorporating relaxation techniques in VR-based treatment for anxiety in autistic adults, which was previously unexplored. Nevertheless, it is crucial to highlight that both investigations featured a notably limited number of participants and did not conduct a comparative analysis of results between the cohort receiving the intervention and the control group.

### Considerations for future research in VR social skills training

Future research, educational programs, and therapeutic measures should prioritize the integration of anxiety management as a pivotal component within the framework of social skills training. The omission of emotional regulation from social skills training could impede the program’s efficacy, given that it constitutes a substantial impediment for individuals on the autism spectrum in their socialization endeavors [38].

Another important aspect is to explore the social steps for initiating, joining, and maintaining a conversation which are relevant and in accordance with the youth. This is called the *ecologically valid social skills steps*—which means the steps taught for social skills training should be pragmatic, practical, and appropriate such as those used by socially successful youths [40].

Considering cultural context could also be beneficial since social skills are influenced by cultural variations. For instance, a study modified the PEERS® model for Dutch culture and observed different reactions to gossip in Dutch and American adolescents. Americans appeared surprised, questioning the significance of gossip, while the Dutch remained indifferent, displaying no reaction [49]. These cultural distinctions emphasize the necessity for developing culturally sensitive VR-based social skills training for diverse populations. This underscores the importance of future research focusing on creating culture and norm-specific VR social skills training programs.

Future research should also put more effort in prioritizing the authenticity of the real-world image that it tries to simulate, especially in relation to non-verbal gestures such as facial expressions. This comes at the light of Parsons’ [30] argument of the extent to which the 3-D images and the immersive characteristics of VR could really represent the real-world situation, considering that social skills require very specific verbal and non-verbal gestures (e.g.: smile, eye contact, smirking). The recent emergence of SVVR interventions for ASD, which relies on 360-degree videos to generate the virtual environment with relatively less complex and cost-effective development [43] seems to provide a promising solution to increase authenticity of the virtual environment and characters.

Another direction of future research is the feasibility and effectiveness of the training when cognitive and behavioral techniques are instantiated in VR environment. These techniques have been well documented as effective in physical social skills training [23, 41, 50], but whether it is feasible and effective when instantiated in VR remains a central question, though previous studies indicate promising results [8, 46, 48].

### Study limitations

There are several limitations to this research. The Phase 1 scientific literature review was constrained to examining social skills challenges and cognitive behavioral techniques that were already employed in VR, potentially neglecting the potential of the specific challenges and techniques that has yet to be implemented within the VR context. Other limitations include the methodology of fuzzy delphi method itself. When expert opinions are represented by fuzzy numbers, there is a lack of reasoning behind the scores, and it could lead to inaccuracy in decision-making. Loss of information could also occur when only distance measures are used to assess the weight and value of opinions between experts [51].

## Conclusion

This study has established a validated framework on the design of social skills training using virtual reality for HFASD. This framework plays a significant role in the development of the training program as it provides standardization by reducing ambiguity, diversity, and discrepancy of opinions, and enhances the quality of selected elements to be included in the training module. It also provides guidance in developing a module that meets the specific needs of the targeted users.
